# Physics-Aware and Intention-Enhanced Trajectory Prediction for Non-Towered Terminal Airspace

**DOI:** 10.3390/s26185725

**Published:** 2026-09-09

**Authors:** Linna Ji, Fengbao Yang

**Affiliations:** School of Information and Communication Engineering, North University of China, Taiyuan 030051, China

**Keywords:** trajectory prediction, spatial–temporal graph attention, flight intention perception, non-towered terminal airspace

## Abstract

To address the challenges in multi-modal trajectory prediction for multi-aircraft interactions within non-towered terminal airspace, including the insufficient extraction of long-range temporal dependencies, neglect of physical separation constraints, and barriers to integrating flight intentions and multi-source environmental context, this paper develops a trajectory prediction model integrated with long-range temporal modeling, physics-aware spatial interaction, and intention context enhancement. A parameter-shared ST-Transformer temporal encoder is established to capture long-term motion patterns of aircraft via global multi-head self-attention, and temporal attention pooling is adopted to mitigate error accumulation in long-term prediction. A physical distance-aware ST-GAT module is designed, which embeds the spatial distance prior between aircraft into the attention calculation and leverages distance masks to reduce interference from distant irrelevant aircraft. Furthermore, an intention-aware context enhancement module (IACEM) is proposed. It identifies the distribution of flight phases and adaptively incorporates meteorological information to construct enhanced features embedded with high-level semantics and environmental priors. Finally, a CVAE-based framework is utilized to generate multiple candidate trajectories satisfying kinematic constraints. Multiple verification experiments are carried out on the TrajAir dataset. The experimental results demonstrate that the proposed model outperforms various baseline models in terms of ADE and FDE. Ablation studies and visual analysis verify that the three core modules produce synergistic improvements. The model achieves higher prediction accuracy under scenarios involving 2D complex maneuvers, 3D climbing turns, and dense multi-aircraft interactions, which proves the effectiveness and superiority of the proposed algorithm for trajectory prediction in non-towered terminal airspace.

## 1. Introduction

With the rapid expansion of general aviation and low-altitude flight industries, flight activities within non-towered terminal airspace have grown increasingly frequent, forming an indispensable component of China’s civil airspace system. Unlike conventional controlled terminal airspace with active air traffic control, non-towered airspace lacks real-time scheduling and separation management from ground towers. Aircraft mainly rely on self-awareness and self-separation to conduct flights, leading to substantially higher autonomy, randomness, and operational uncertainty [1,2]. Such airspace accommodates diverse aircraft types and frequent transitions between flight phases, where multiple flight behaviors, including approach, departure, and holding, intersect with each other. Dense aircraft interactions raise prominent risks of local flight conflicts, imposing severe challenges on safe and efficient airspace management. As a fundamental technology supporting air traffic conflict detection, airspace situational awareness, and autonomous separation assurance, trajectory prediction delivers critical support for stable and secure non-towered terminal airspace operations, conflict risk reduction, and improved utilization of airspace resources [3].

Compared with trajectory forecasting tasks in conventional airspace, multi-aircraft trajectory prediction within non-towered terminal airspace is far more than simple single-agent temporal extrapolation. It represents a complex spatio-temporal forecasting problem governed by coupled factors, intensive agent interactions, and high uncertainty. From the perspective of trajectory evolution, aircraft flight paths exhibit strong temporal correlation and spatial interdependence. In the temporal dimension, historical flight states continuously constrain subsequent trajectory trends. Spatially, flight behaviors of different aircraft mutually restrict one another, and separation requirements directly shape trajectory decision-making for individual aircraft [4]. From environmental and intentional perspectives, external heterogeneous factors such as meteorological disturbances and airspace operational status, together with high-level semantic information including pilot maneuvers and flight mission plans, further amplify multi-modal uncertainties in trajectory evolution and raise barriers to high-precision trajectory modeling [5]. Accordingly, trajectory prediction for non-towered terminal airspace requires simultaneous breakthroughs in long-range temporal dependency modeling, multi-aircraft physical interaction characterization, and multi-source context fusion.

In recent years, data-driven deep learning has become the mainstream technical route for airspace trajectory prediction. As a representative baseline model for multi-aircraft interactive trajectory prediction in non-towered terminal airspace, TrajAirNet integrates temporal encoding, graph-attention spatial interaction modeling, and conditional variational generation mechanisms. It satisfies basic requirements for multi-agent trajectory forecasting and achieves promising performance in complex terminal airspace scenarios [6]. Nevertheless, the model still suffers from evident limitations when deployed in high-density, highly dynamic, and severely perturbed non-towered terminal environments. For temporal modeling, the TCN-based encoder expands local receptive fields but struggles to capture non-local temporal dependencies over extended horizons. This causes loss of features at critical flight timestamps and trajectory drift during long-sequence forecasting. For spatial interaction modeling, its native graph attention allocates weights merely based on latent feature similarity, without incorporating physical prior knowledge such as relative aircraft distance and safety separation. It thus fails to accurately characterize safe interaction patterns between aircraft and cannot cope with modeling requirements in high-conflict scenarios. For context modeling, heterogeneous information such as weather and airspace status is integrated in a static manner. Meanwhile, flight intention, the high-level semantic factor dominating trajectory variations, is overlooked, impairing the model’s performance for dynamic complex multi-aircraft scenarios under the current airport operational distribution.

Targeting the aforementioned drawbacks of existing approaches, this paper develops a multi-aircraft trajectory prediction model equipped with spatio-temporal graph attention and multi-source intention-aware enhancement, taking TrajAirNet as the baseline. The main contributions of this work are summarized as follows:

(1) A ST-Transformer is developed, which incorporates flight physical priors into spatio-temporal attention and adopts a temporal attention-pooling mechanism to adaptively capture informative historical observations for interactive aircraft. Different from standard Transformer models for trajectory forecasting, physical constraints participate in attention weight computation rather than being merely appended as auxiliary loss terms.
(2) A physics-aware ST-GAT module is proposed. Instead of using a fixed graph topology, it dynamically builds adjacency relationships according to real-time spatial distance between aircraft, embedding air-traffic separation constraints inside graph attention operations.
(3) An intention-aware context enhancement module IACEM is designed to infer implicit multi-aircraft flight intentions without requiring explicit destination priors, which distinguishes it from existing goal-conditioned trajectory prediction methods.

These modules are assembled upon the CVAE-based multi-modal generation backbone from TrajAirNet, yet each module delivers targeted methodological improvements for complex multi-aircraft interaction under non-towered airport conditions. Coordinated multi-dimensional optimizations effectively remedy modeling defects of the baseline model, considerably boosting prediction accuracy and robustness for multi-aircraft trajectories in complex non-towered terminal airspace. This work provides solid technical support for intelligent traffic management and flight conflict early warning within low-altitude non-towered airspace.

## 2. Related Work

Flight trajectory prediction models future aircraft motion based on historical trajectory observations. As a core technology in air traffic management (ATM), it provides essential support for airspace situational awareness, conflict detection, and operational safety assurance. Existing trajectory prediction approaches can be categorized into state estimation methods, dynamics-driven modeling methods, and data-driven methods according to their modeling principles.

### 2.1. State Estimation-Based Trajectory Prediction Methods

These methods abstract aircraft movement as a temporal state evolution system. On the basis of filtering, probability statistics, and fitting theories, historical observations are adopted to establish state recursion models for estimating flight states such as position, velocity, and acceleration. Representative approaches include the Kalman filter and its variants [7,8], Hidden Markov models [9], least squares fitting [10], and interacting multiple model (IMM) [11,12]. Current studies improve estimation accuracy by means of unscented transformation, polynomial fitting, weather-associated modeling, and multi-model matching, addressing challenges such as missing data, environmental disturbances, and flight mode transitions. Generally speaking, state estimation methods feature simple architectures and perform well under regular flight scenarios, yet they have inherent limitations. Aircraft motion exhibits strong randomness under high-speed maneuvers and severe meteorological disturbances, making it difficult to formulate accurate and fixed state evolution equations. In addition, such methods usually suffer from heavy computation and response latency, leading to insufficient real-time performance and robustness within dynamically complex airspace.

### 2.2. Dynamics-Based Trajectory Prediction Methods

Belonging to mechanism-driven modeling, this category relies on aircraft force analysis and physical motion laws. Combined with prior information including BADA aircraft performance parameters, meteorological conditions, and flight intentions, full-phase trajectories are derived by solving dynamic differential equations [13,14]. Existing research improves modeling accuracy for typical phases such as climb and cruise via wind field correction, aircraft mass error optimization, fusion of meteorological and intent information, and dynamic real-time parameter calibration. Such methods deliver clear physical interpretability and enable refined modeling subject to aircraft performance and airspace environmental constraints. Nevertheless, notable drawbacks remain. The models involve high dimensionality and complicated aerodynamic constraints, and parameter calibration heavily relies on engineering experience, which increases modeling difficulty. Furthermore, numerical solutions bring considerable computational overhead, making it hard to balance prediction accuracy and real-time performance in highly dynamic, disturbed terminal airspace.

### 2.3. Data-Driven Trajectory Prediction Methods

Instead of requiring precise physical mechanisms, these methods discover motion patterns from massive historical trajectory datasets and establish non-linear mappings between input features and future trajectories. Many existing works adopt CNN [15,16], LSTM [17,18], spatial–temporal graph convolution [19,20], GAN, and hybrid architectures [21,22] to boost prediction performance from the perspectives of temporal feature extraction, spatial correlation modeling, and adversarial optimization. Several studies introduce attention mechanisms to strengthen the weights of critical features and refine trajectory prediction results. Compared with conventional algorithms, deep learning models possess a stronger capability to fit complex scenarios and capture non-linear relationships. Even so, prevailing data-driven approaches still have obvious shortcomings. Most models focus merely on temporal modeling and fail to adequately characterize the spatio-temporal coupling among multi-dimensional flight parameters. When handling dense traffic, multi-aircraft interactions, and frequent maneuvers in terminal airspace, they struggle to precisely model spatial restrictions and dynamic interactions between aircraft. Moreover, high-level semantic information such as flight intentions and dynamic environmental conditions is insufficiently exploited. Prediction stability during critical flight phases and generalization ability across complex scenarios need further improvement.

Despite considerable progress achieved in recent trajectory-prediction studies, several technical gaps remain for multi-aircraft prediction under non-towered-airport conditions. Transformer-based flight prediction models leverage global self-attention to capture temporal dependencies, yet physical flight knowledge is typically introduced only through auxiliary loss functions, instead of participating in attention weight calculation. Different from these approaches, our ST-Transformer incorporates flight-physical priors within spatio-temporal attention, accompanied by a temporal attention-pooling module to adaptively highlight informative historical segments for multi-agent interactions. Graph-attention-based aircraft prediction methods model aircraft interactions via graph structures; however, most rely on fixed predefined topology. Our distance-aware ST-GAT constructs adjacency relationships dynamically according to real-time spatial distance, and embeds air-traffic separation constraints directly into graph-attention computation, rather than constraining prediction outputs via standalone loss penalties. Existing physics-constrained forecasting methods commonly apply physical rules as post-processing regularization upon predicted trajectories. In our framework, physical awareness acts inside feature encoding, guiding the learning of interactive representations. For intention-aware trajectory prediction, many recent goal-conditioned approaches require pre-known or pre-estimated flight destinations as model inputs. Such priors are hard to obtain for non-towered-airport operations, where flight intentions may dynamically shift for collision avoidance. Our IACEM infers implicit multi-aircraft interactive intentions without explicit destination priors. Environment-aware trajectory models usually rely on manually encoded static airport semantic maps. By contrast, our dynamic graph mechanism implicitly captures local airspace interaction patterns, removing the requirement for hand-crafted environmental masks.

To tackle the deficiencies of existing models, including inadequate coupled spatio-temporal feature modeling, lack of physical constraints, and weak intention perception, this paper proposes a trajectory prediction model integrated with spatio-temporal graph attention and multi-source intention awareness, aiming to achieve higher accuracy and stability for trajectory prediction in complex terminal airspace.

## 3. The Proposed Method

**Figure 1:**
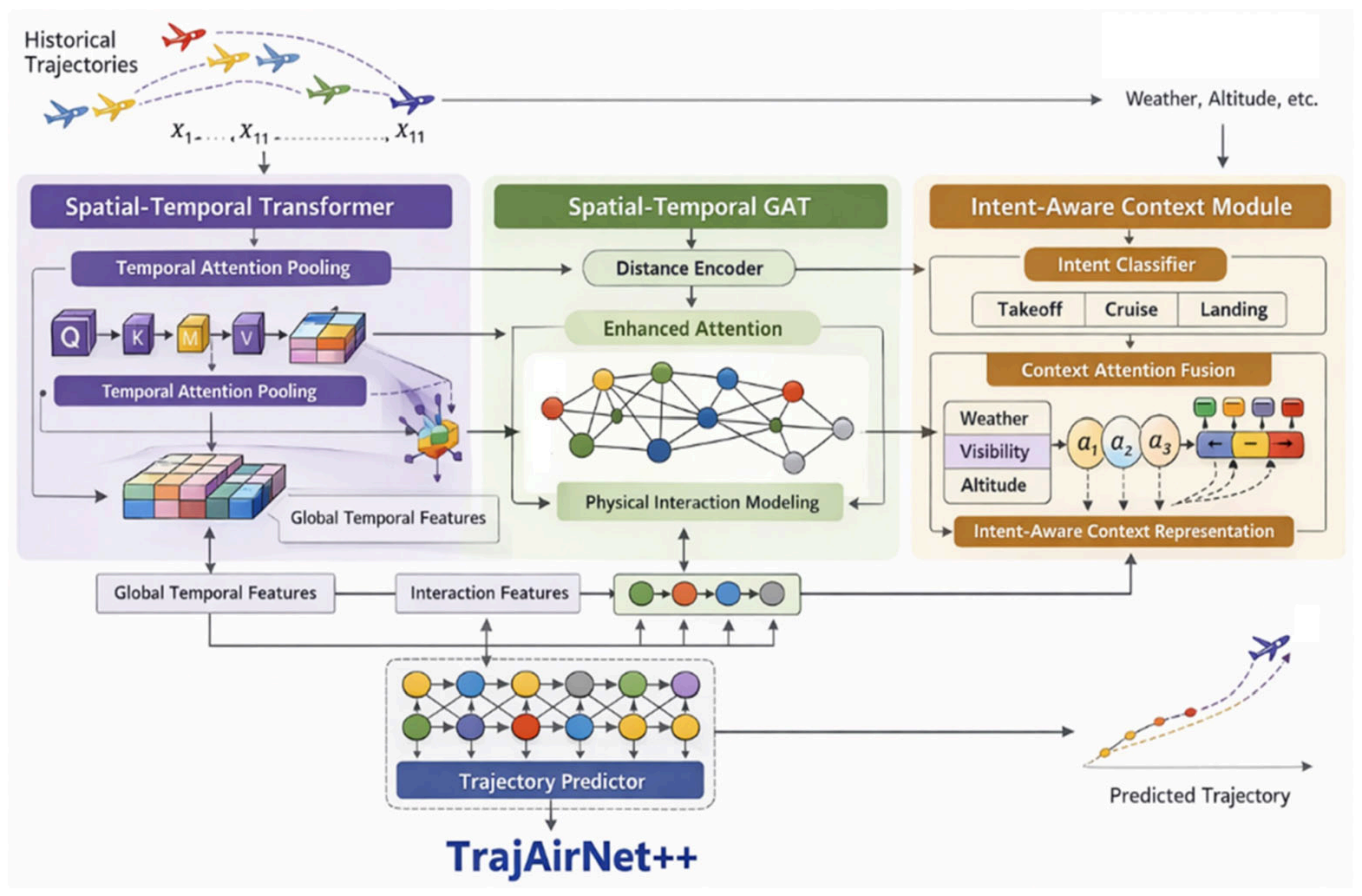
The overall architecture of the proposed model.

Trajectory evolution of multiple aircraft within non-towered terminal airspace is governed jointly by long-range temporal dependencies, spatial interactions among aircraft, and external environmental disturbances. Targeting multi-aircraft interactive trajectory prediction in such airspace, this paper conducts an integrated collaborative design covering temporal feature extraction, spatial interaction modeling, context fusion, and trajectory generation. The overall architecture of the proposed model is illustrated in Figure 1, which consists of four core components: a temporal feature encoding module, a physics-aware spatial–temporal interaction modeling module, an intention-aware dynamic context fusion module, and a CVAE-based multi-modal trajectory generation module embedded with kinematic constraints.

From the perspective of data flow, the proposed model establishes an integrated end-to-end prediction pipeline covering long-range temporal feature learning, multi-source context and intention enhancement, physically constrained spatial interaction modeling, and controllable multi-modal trajectory generation. Specifically, the ST-Transformer paired with temporal attention pooling mitigates insufficient long-sequence temporal modeling and loss of critical temporal information. The ST-GAT module focuses on characterizing physical multi-aircraft interactions, overcoming the drawback that conventional latent-space interaction modeling ignores physical airspace constraints. IACEM improves generalization in complex operating scenarios by integrating heterogeneous environmental data and flight status features. The kinematics-constrained CVAE generation module not only captures multi-modal uncertainty in future trajectory evolution, but also guarantees the physical plausibility and kinematic feasibility of predicted flight paths.

### 3.1. ST-Transformer Module

**Figure 2:**
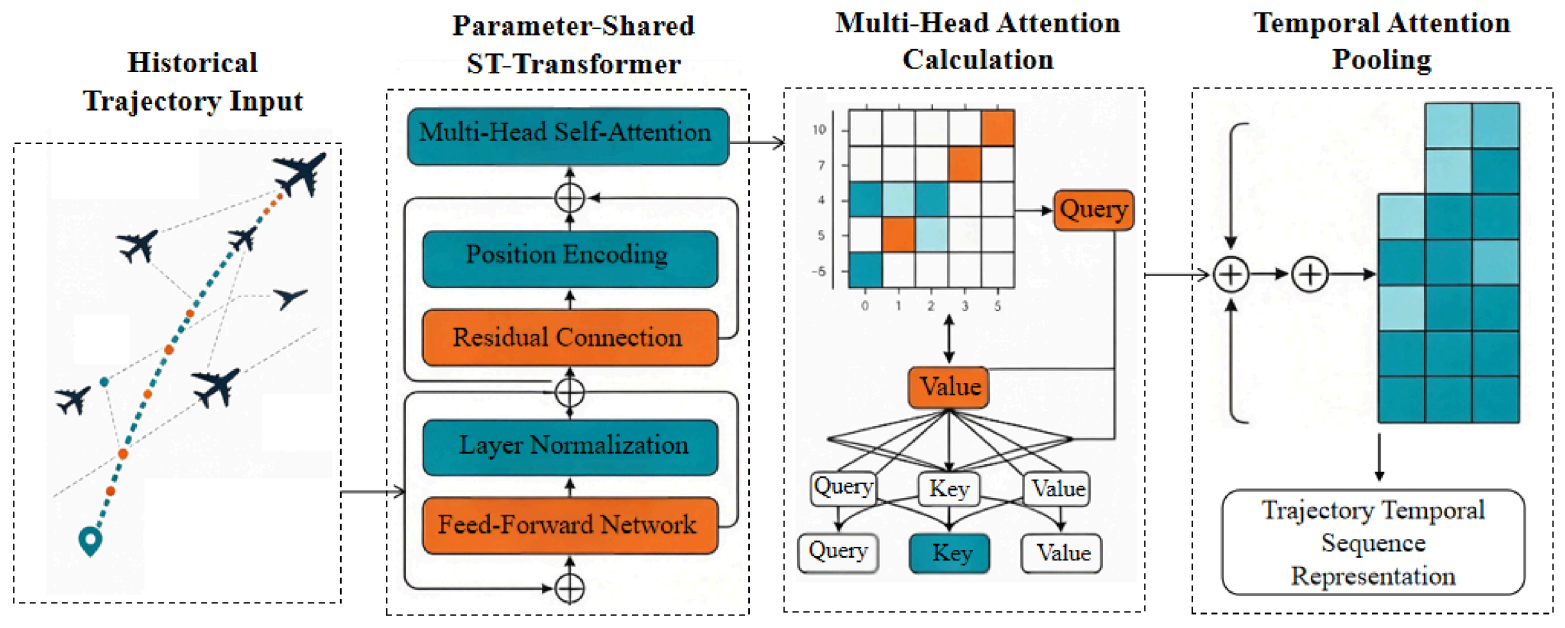
ST-Transformer architecture.

Aircraft operations within non-towered terminal airspace are characterized by high traffic density, frequent complex maneuvers, and temporally cumulative trajectory evolution. The future trajectory of an aircraft is not solely determined by instantaneous motion parameters; instead, it arises from the cumulative coupling of sequential historical flight behaviors. Typical maneuvers, including speed reduction during approach, climb after departure, speed adjustment in holding patterns, and heading corrections, exhibit obvious temporal lag effects and behavioral continuity. Early flight decisions continuously shape the evolutionary tendency of trajectories over a long subsequent period. Accordingly, effectively capturing cross-time correlations embedded in long trajectory sequences and accurately identifying critical temporal segments that dominate future traffic conditions constitute a key technical challenge for high-precision multi-aircraft interactive trajectory prediction. The architecture of the ST-Transformer is illustrated in Figure 2.

Among existing temporal modeling solutions, the temporal convolutional network (TCN) is widely adopted for short-sequence trajectory fitting owing to its support for parallel computation and capability to capture local temporal patterns [23,24]. TCN expands its receptive field by stacking dilated convolutions with fixed kernel sizes. It can only model local dependencies between adjacent timestamps within limited intervals, and inherently lacks the ability to globally characterize long-range temporal correlations. In terminal airspace, flight maneuvers typically span dozens of observation steps. Subsequent adjustments of altitude, speed, and heading are constrained by previous flight states. The inherent limitation of local modeling in TCN leads to loss of long-distance temporal connections and continuous accumulation of long-term prediction errors, making it inadequate for modeling long trajectory sequences in complex terminal airspace.

To remedy the insufficient modeling of long-range dependencies in TCN, this paper proposes a parameter-shared Spatial–Temporal Transformer (ST-Transformer) to conduct independent temporal encoding for each aircraft. The global self-attention mechanism breaks the restriction of local convolutional receptive fields, directly establishing dynamic connections across all timestamps and fully capturing global long-range temporal dependencies embedded in trajectory sequences. Furthermore, the parameter-sharing strategy is deployed to encode historical sequences of all aircraft uniformly. While maintaining consistent temporal feature distributions among multiple aircraft, this scheme effectively reduces model parameters and inference overhead. Its scaled dot-product attention is formulated as:

(1) $$A_{ttn}(Q,K,V)=\mathrm{softmax}(\frac{QK^{T}}{\sqrt{d_{k}}})V$$

where *Q*, *K,* and *V* denote the Query, Key, and Value matrices, respectively. $d_{k}$ refers to the dimension of Key vectors, and $\sqrt{d_{k}}$ is a scaling factor to avoid excessively large inner products that would drive the softmax function into regions with vanishing gradients.

To enable parallel extraction of multi-aspect temporal correlations, multi-head self-attention employs multiple independent linear projections to capture temporal features within distinct representation subspaces. Its mathematical formulation is:

(2) $$\mathrm{MultiHead}(Q,K,V)=\mathrm{Concat}(head_{1},head_{2},\ldots ,head_{h})W^{O}$$

where the computation of the i-th attention head is expressed as:

(3) $$head_{i}=Attention(QW_{i}^{Q},KW_{i}^{K},VW_{i}^{V})$$

Different from vanilla multi-head attention, we introduce a flight-physical prior modulation matrix $M_{phys}$ to regularize raw attention weights before softmax normalization. $M_{phys}$ is constructed from spatial proximity and kinematic consistency priors of flight states, which suppresses spurious attention responses between physically irrelevant aircraft–timestamp pairs and strengthens weights for physically plausible interaction segments. The physically modulated attention for each head becomes:

(4) $$head_{i}=softmax\left(\frac{QW_{i}^{Q}{\left(KW_{i}^{K}\right)}^{T}}{\sqrt{d_{k}}}⊙M_{phys}\right)(VW_{i}^{V})$$

where ⊙ denotes the element-wise Hadamard product. Entries in $M_{phys}$ take smaller values for physically disconnected flight-time pairs and approach 1 for kinematically and spatially correlated segments. $W_{i}^{Q}$, $W_{i}^{K}$, and $W_{i}^{V}$ are learnable projection matrices belonging to the i-th head, and $W^{O}$ represents the output projection matrix. Benefiting from parallel multi-head modeling, the model is capable of capturing temporal correlations within trajectory sequences from diverse perspectives, lifting its capacity to represent complex dynamic variations.

Since each historical time step contributes differently to future trajectory prediction, we equip the output sequence of ST-Transformer with a temporal attention pooling module. The module performs adaptive weighted aggregation over temporal information and automatically emphasizes vital historical segments that govern later prediction. Suppose the encoded sequential feature is denoted as $Z=\left\{z_{1},z_{2},\ldots ,z_{T}\right\}$, and $z_{t}$ represents the feature vector at the t-th observation moment. The formulation is written as:

(5) $$s_{t}=W_{2}^{T}(W_{1}z_{t}+b_{1})+b_{2}$$

(6) $$h^{traj}=\sum_{t=1}^{T_{f}}\frac{\exp(s_{t})}{\sum_{\tau =1}^{T_{f}}\exp(s_{\tau })}z_{t}$$

where $W_{1}$ and $W_{2}$ represent trainable weight matrices, and $b_{1}$ and $b_{2}$ correspond to bias vectors. Here, σ(⋅) denotes the non-linear activation function applied to raw attention scores before weight normalization. The weighted fusion of timestamp-wise features enables the model to preserve global temporal context and prioritize historical segments critical to upcoming trajectory changes. In this way, the temporal embedding for each aircraft trajectory is generated. After iterating over all aircraft in the airspace, we acquire a collection of temporal features $\left\{h_{1}^{traj},h_{2}^{traj},\ldots h_{N}^{traj}\right\}$, which are fed into the succeeding module for multi-aircraft spatial interaction modeling.

It is worth noting that this pipeline is not a trivial combination of vanilla Transformer and generic attention pooling. Different from common sequence forecasting implementations, flight-physical priors are embedded inside spatio-temporal attention calculation, instead of being added only as an auxiliary loss. Moreover, the temporal attention-pooling module is tailored for non-towered-airport multi-aircraft scenarios: it adaptively identifies and emphasizes historical time segments associated with potential collision-avoidance maneuvers, rather than simply compressing the complete sequence into a fixed-dimensional vector. The resulting temporal features are further fed into the downstream graph module to support multi-agent interaction reasoning.

### 3.2. Horizontally Physics-Aware Graph Attention Module

Traditional graph models build connections only through latent features and neglect physical separation constraints within airspace. To overcome this drawback, we construct the spatial–temporal graph attention (ST-GAT) module. Spatial distance priors between aircraft are explicitly introduced to improve the capacity of the model to characterize physical rules, including safety separation and intensive close-range interactions in terminal airspace.

The standard graph attention network (GAT) calculates cross-node attention weights purely based on latent feature similarity. Though it can discover potential connections between aircraft, such a mechanism operates without considering physical air traffic rules and cannot adapt to terminal airspace scenarios. Under practical air traffic management regulations, the intensity of aircraft interactions is strongly negatively correlated with physical separation. The closer two aircraft are, the higher the collision risk and the stronger mutual restrictions on their maneuvers. By contrast, distant aircraft barely affect the trajectory decision-making of the target aircraft. If distance-based physical priors are discarded and attention weights are assigned only according to feature similarity, irrelevant distant aircraft may receive excessive interaction weights, while critical nearby aircraft fail to gain sufficient representation. This issue substantially degrades the accuracy of multi-aircraft interaction modeling.

We integrate physical distance information into graph attention to establish a dual-perception attention computation mechanism combining latent feature similarity and spatial constraints. For the m-th attention head, the attention score between node *i* and node *j* is defined as:

(7) $$e_{ij}^{\left(m\right)}=\mathrm{LeakyRelu}(a_{s}^{{\left(m\right)}^{T}}[W_{s}^{\left(m\right)}h_{i}\left\|W_{s}^{\left(m\right)}h_{j}\right.])+\varphi (d_{ij})$$

where *h_i_* and *h_j_* denote the input features of node *i* and node *j*, respectively. $W_{s}^{\left(m\right)}$ is the linear projection matrix of the m-th attention head, and $a_{s}^{\left(m\right)}$ stands for the corresponding learnable attention parameter. || represents vector concatenation. $d_{ij}$ is the physical distance between aircraft *i* and *j*, and ϕ(⋅) refers to the distance encoding function that maps distance information into attention calculation. $\varphi (d_{ij})=\frac{1}{1+\alpha \cdot d_{ij}}$ where α=0.2 is a fixed scaling hyperparameter. This mapping suppresses the attention weight for spatially far-apart aircraft and amplifies the influence of nearby interactive agents, which is consistent with physical airspace separation constraints.

With this design, the model leverages feature similarity while explicitly accounting for spatial adjacency among aircraft. Furthermore, to mitigate interference from irrelevant nodes, an adjacency matrix *A* constructed via distance thresholds is adopted to impose masking constraints on attention values, formulated as:

(8) $$e_{ij}^{m}\leftarrow \left\{\begin{array}{c} e_{ij}^{\left(m\right)},A_{ij}=1\\ -\infty ,A_{ij}=0 \end{array}\right.,\;\alpha_{ij}^{\left(m\right)}={\mathrm{softmax}}_{j}(e_{ij}^{\left(m\right)})$$

where $A_{ij}=1$ the indicator means node *j* lies within the valid interaction neighborhood of node *i*; otherwise, no direct interaction is established between them. Benefiting from the masking strategy, the model focuses attention on neighboring aircraft with tight correlations to the target aircraft and suppresses disturbances caused by distant or weakly related nodes. After interaction modeling, a set of interaction features $\left\{h_{1}^{soc},h_{2}^{soc},\ldots ,h_{N}^{soc}\right\}$ can be obtained. These features integrate positional information, motion restrictions of surrounding aircraft, and physical priors of airspace safety separation. Together with the previously derived intention-aware enhanced features, they are fed into the subsequent multi-modal trajectory generation module, providing comprehensive spatial interaction conditions for prediction outputs.

In this work, the physical prior adopted in ST-GAT is mainly built upon horizontal Euclidean separation, which serves as the primary condition for triggering mutual interaction for aircraft operating within non-towered airport traffic patterns. Pairs of aircraft whose horizontal distance exceeds a pre-defined threshold are disconnected from the graph, as their mutual interactive influence is regarded as negligible. This spatial threshold is selected according to airport separation regulations and empirical statistics of the TrajAir dataset, and is tuned on the validation set to prevent test-set data leakage. We note that the current implementation does not incorporate relative velocity, heading, and vertical motion cues into graph topology construction, which leaves room for further improvement of conflict-risk modeling.

### 3.3. Intention-Aware Context Enhancement Module

Aircraft trajectory evolution in terminal airspace is governed by complex coupled factors, including historical motion patterns, multi-aircraft spatial interactions, meteorological disturbances, and phase-dependent operational constraints. Specifically, wind fields, visibility conditions, airport arrival/departure traffic flow, and runway scheduling significantly restrict aircraft speed adjustment, heading variation, and altitude variation strategies. Flight behaviors differ substantially across operational phases: aircraft primarily perform descent adjustment and runway alignment during the approach phase, while separation maintenance and traffic flow compliance dominate cruising and holding maneuvers. Pure trajectory-based temporal models cannot capture these phase-variant environmental and operational constraints, leading to limited generalization ability for complex terminal trajectory prediction.

**Figure 3:**
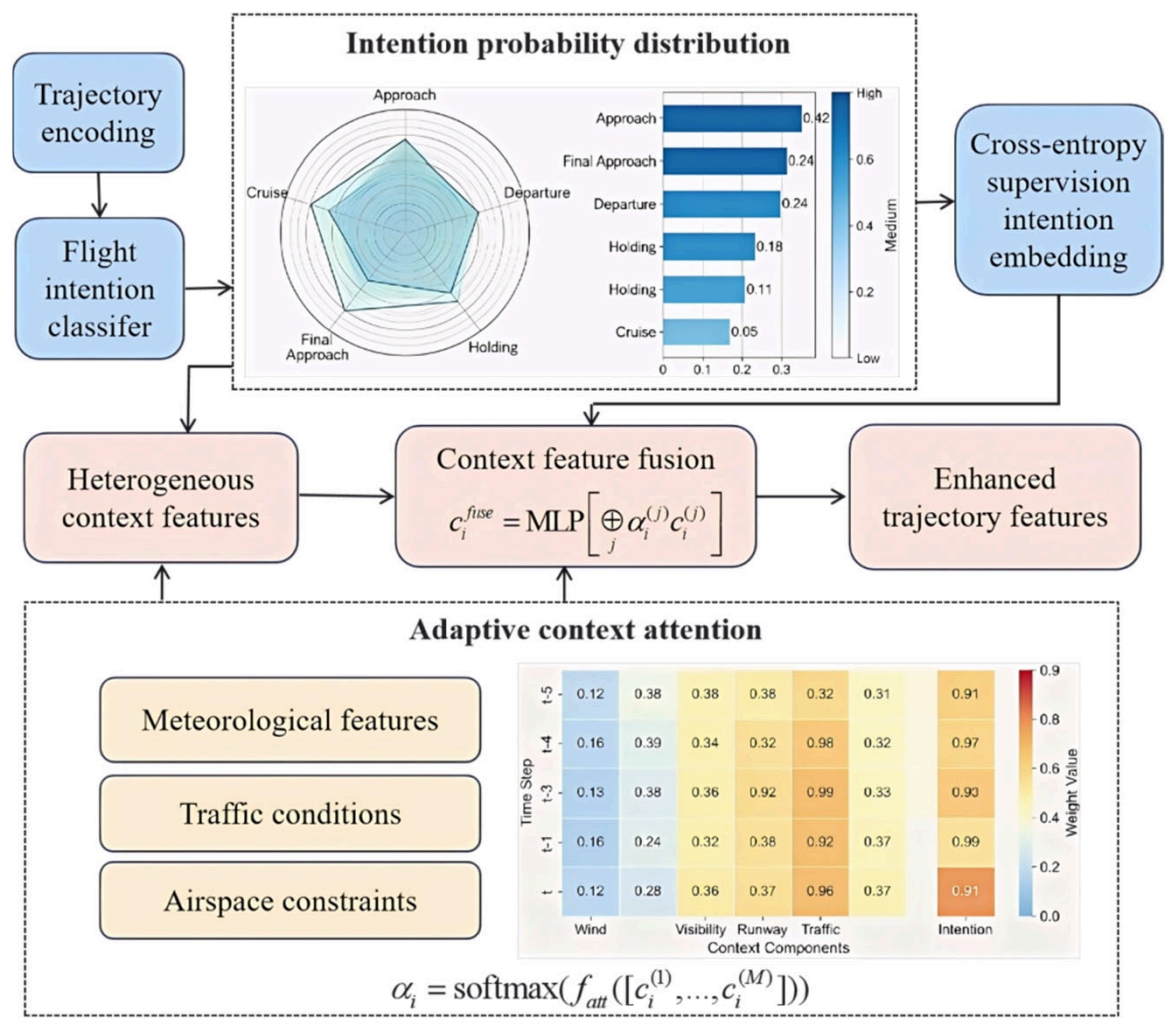
Flowchart of intention-aware context enhancement.

To address the insufficient modeling of multi-source heterogeneous context and flight operational semantics, we propose the intention-aware context enhancement module (IACEM), as shown in Figure 3. This module infers flight operational states from historical kinematic sequences and adaptively fuses multi-dimensional contextual information, producing semantic-enhanced trajectory features conditioned on real-time flight intentions and environmental priors.

Different from studies relying on manually annotated flight-intention labels, this work derives kinematics-based pseudo-intention supervision automatically from raw ADS-B observations. No human-labeled semantic intention or flight-phase annotation is adopted. In this work, discrete intention pseudo-labels are automatically generated frame-by-frame based on ADS-B kinematic parameters without manual annotation. We define three core flight maneuver states according to physical motion characteristics in terminal airspace: (1) Level Flight: low vertical rate (|vertical speed| < 0.5 m/s) and stable heading; (2) Climb/Descend: significant vertical rate (|vertical speed| > 0.5 m/s); and (3) Turning Motion: high heading change rate (|heading derivative| ≥ 0.3°/s). These threshold values are empirically determined according to the flight characteristics of general aviation aircraft in non-towered terminal scenarios. All trajectory frames are automatically assigned to one of the three kinematic maneuver categories, forming discrete pseudo-labels for auxiliary cross-entropy supervision. These data-driven discrete maneuver states serve as pseudo-ground-truth categories for auxiliary intention learning.

Based on the temporal embeddings encoded from flight sequences, the network predicts the probability distribution over these discrete kinematic maneuver categories. The intention inference process is formulated as:

(9) $$g_{i}=\arg\max p_{i},p_{i}=\mathrm{softmax}(f_{intent}(h_{i}^{traj}))$$

where $f_{\mathrm{int}ent}(\cdot )$ denotes the intention recognition function, $g_{i}$ stands for the intention classification result of aircraft *i*, and $p_{i}$ represents the corresponding intention probability distribution. Through this auxiliary classification task, the model automatically captures high-level flight operational semantics (e.g., approaching, climbing, turning, holding) hidden in trajectory variations, which provides phase-aware semantic prior knowledge for subsequent context fusion. The discrete flight-phase illustrations in Figure 3 visually correspond to these kinematically partitioned maneuver categories used for model supervision.

Once intention information is obtained, aircraft intention features $c_{i}^{intent}$, meteorological data $c_{i}^{weather}$ and other contextual signals are all fed into a unified fusion workflow, producing context embeddings that match each aircraft’s real-time flight status. Since environmental factors exert uneven influence across different flight phases, an adaptive context attention module is adopted to dynamically calculate the importance weight of every contextual element. Suppose $c_{i}^{\left(1\right)},c_{i}^{\left(2\right)},\ldots c_{i}^{\left(M\right)}$ is the raw context feature of aircraft *i*, the weight of each contextual component is expressed as:

(10) $$\alpha_{i}=\mathrm{softmax}(f_{att}([c_{i}^{\left(1\right)},\ldots ,c_{i}^{\left(M\right)}]))$$

Here, $f_{att}(\cdot )$ is the context weight calculation function, and $\alpha_{i}$ represents normalized weights for all categories of contextual information. Using these weights, multi-source context features are aggregated into a unified embedding through the following formula:

(11) $$c_{i}^{fuse}=\mathrm{MLP}\left[\underset{j}{⊕}\alpha_{i}^{\left(j\right)}c_{i}^{\left(j\right)}\right]$$

where MLP(⋅) refers to the non-linear mapping network for cross-source feature integration, while $c_{i}^{fuse}$ stands for the integrated context embedding of aircraft *i*. The original temporal features $h_{i}^{traj}$, intention embeddings $g_{i}$ and fused context features $c_{i}^{fuse}$ of each aircraft are concatenated, followed by one linear projection layer to unify feature dimensions. The final output is the aircraft’s intention-aware enhanced feature, defined as:

(12) $${\tilde{h}}_{i}=W\cdot \mathrm{Concat}(h_{i}^{traj},g_{i},c_{i}^{fuse})+b$$

This embedding integrates three layers of information: evolution patterns extracted from historical flight sequences, high-level flight intention semantics including approach and departure, and multi-source environmental constraints involving weather and airspace operation conditions. It remedies insufficient information when modeling only with motion sequences, and provides complete conditional inputs for later physically constrained multi-aircraft spatial interaction modeling and multi-modal trajectory generation.

It is worth noting that no manually annotated discrete flight-intention ground-truth labels are available within the TrajAir dataset, and no manual annotation is performed in this work. Instead, the intention-aware context enhancement module adopts implicit supervisory signals derived purely from raw ADS-B trajectory kinematics. Heading variation rate, acceleration magnitude, and pairwise relative motion between aircraft are computed as rule-driven cues to reflect ongoing turning, speed adjustment, and collision-avoidance cooperative maneuvers. Our framework does not define a fixed set of discrete intention classes; flight intention is represented as continuous implicit context features learned from these trajectory-derived motion cues.

### 3.4. Multi-Modal Trajectory Generation Module

Aircraft future flight paths contain inherent stochastic uncertainty. Even under identical historical observation data, multiple physically reasonable trajectories consistent with flight rules and kinematic characteristics can be generated. We concatenate intention-enhanced temporal embeddings ${\tilde{h}}_{i}$ and spatial interaction features $h_{i}^{soc}$ of each aircraft as the input to a conditional variational autoencoder (CVAE), so as to predict future motion control values. The prediction target is defined as discrete-time acceleration sequences ${\hat{a}}_{i}$, from which complete future flight paths ${\hat{y}}_{i}$ can be recovered via kinematic relations. For every discrete time step t in the prediction window, the network outputs aircraft acceleration control quantities ${\hat{a}}_{i,t}$. We adopt second-order discrete kinematic iterative formulas to update instantaneous velocity and global spatial coordinates step by step, as shown in Equations (13) and (14):

(13) $${\hat{v}}_{i,t}={\hat{v}}_{i,t-1}+{\hat{a}}_{i,t}\Delta t$$

(14) $${\hat{y}}_{i,t}={\hat{y}}_{i,t-1}+{\hat{v}}_{i,t-1}\Delta t+\frac{1}{2}{\hat{a}}_{i,t}\Delta t^{2}$$

Δt represents the discrete prediction time interval; ${\hat{v}}_{i,t-1}$ and ${\hat{y}}_{i,t-1}$ separately denote the predicted velocity and absolute position of the aircraft at the previous time step.

To realize fully differentiable end-to-end joint training across the temporal encoder, intention enhancement module, spatial interaction network, and multi-modal decoder, we design a multi-task composite loss function constrained by three loss items: trajectory reconstruction loss, KL divergence regularization loss, and flight intention cross-entropy classification loss L:

(15) $$L=\lambda_{traj}L_{traj}+\lambda_{KL}L_{KL}+\lambda_{intent}L_{intent}$$

$L_{traj}$ is the trajectory reconstruction loss, which restricts the coordinate sequence generated by the decoder to be consistent with ground-truth trajectories. Its computational formula is:

(16) $$L_{traj}=\frac{1}{NT_{f}}\sum_{i=1}^{N}\sum_{t=1}^{T_{f}}||{\hat{y}}_{i,t}-y_{i,t}|{|}_{2}$$

*N* refers to the total number of aircraft within the airspace scene, and $y_{i,t}$ is the real spatial coordinate of the i-th aircraft at time t. $L_{KL}$ stands for the KL divergence regularization term. It constrains the latent distribution learned by the CVAE encoder to approach the standard Gaussian distribution, maintaining the model’s ability to sample diverse multi-modal trajectories, with the formula as follows:

(17) $$L_{KL}=\frac{1}{N}\sum_{i=1}^{N}\frac{1}{2}\sum_{k=1}^{d_{z}}\left(\mu_{i,k}^{2}+\exp(\log\sigma_{i.k}^{2})-1\right)$$

$\mu_{i,k}$ and $\log\sigma_{i,k}^{2}$ are the mean and logarithmic variance of latent features output by the encoder, $d_{z}$ is the dimension of the latent space. $L_{intent}$ is the flight intention classification loss adopting cross-entropy loss, which supervises the model’s prediction of aircraft operation phases:

(18) $$L_{intent}=-\frac{1}{N}\sum_{i=1}^{N}\sum_{k=1}^{K}I(g_{i}=k)\log p_{i}(k)$$

where *λ_traj_* is fixed to 1.0 to prioritize the primary trajectory generation task, which serves as the core optimization objective. To balance the magnitude of different loss terms and avoid training dominance or vanishing issues, we determine the remaining hyperparameters via extensive grid-search ablation experiments on the validation set, searching *λ_KL_* within [0.05, 0.2] and *λ_intent_* within [0.1, 0.5]. The optimal configuration is identified as *λ_KL_* = 0.1 and *λ_intent_* = 0.2 according to the minimal minADE. Specifically, a relatively small *λ_KL_* alleviates the KL-vanishing problem and prevents the regularization term from suppressing trajectory reconstruction, while a moderate *λ_intent_* ensures effective auxiliary supervision for multi-aircraft interactive modeling without overriding the main trajectory-fitting task. Moreover, a KL-annealing strategy is adopted during the first 50 training epochs to further stabilize CVAE latent-space optimization.

Overall, the composite multi-objective loss realizes triple supervision: prediction coordinate precision, latent space distribution regularization, and high-level flight intention semantic constraint. It guides the model to capture spatio-temporal interaction evolution rules of multiple aircraft, while accurately modeling aircraft high-level flight intentions and multi-modal trajectory randomness. The final output trajectories feature high precision, kinematic rationality, and mode diversity, which can adapt to complex dynamic operation scenarios of non-towered terminal airspace.

## 4. Experiments and Result Analysis

### 4.1. Dataset and Preprocessing

The TrajAir dataset serves as the experimental benchmark to evaluate the performance of our proposed model. Designed for multi-aircraft interaction prediction in non-towered terminal airspace, TrajAir is an open dataset containing high-fidelity aircraft trajectory data and synchronized meteorological records. It can accurately reflect the complex multi-source coupling characteristics of trajectory evolution in busy terminal airspace [6]. ADS-B data supplies fundamental motion information of every aircraft, including 3D spatial positions, precise timestamps, and unique aircraft ID tags. To meet numerical simulation requirements, raw geodetic coordinates (longitude, latitude, altitude) are converted into a local Cartesian coordinate system, removing calculation errors induced by spherical coordinate modeling. Corresponding METAR weather data provides airspace environmental parameters precisely synchronized with trajectory timestamps, with core meteorological indicators including 2D wind field components calculated from wind speed and direction. These synchronized METAR records are only utilized as internal context resources within our IACEM and are not provided as external network inputs for baseline models. Such real measured data lays a solid foundation for the model to learn how weather disturbances restrict aircraft maneuver operations and trajectory changes.

Raw flight trajectory data features high sampling rates, which leads to heavy redundancy between adjacent time samples. Consistent with the preprocessing pipeline of baseline algorithms, we adopt sliding window sampling and downsampling strategies. These methods reduce data scale and computational cost while retaining the overall changing trends of flight paths. The specific window settings are as follows: the historical observation window covers 11 time steps, which means the input to the model consists of continuous 11-frame historical trajectories and matched meteorological context information. The full prediction window covers 120 original time steps. We adopt fixed-step sparse sampling to select 12 critical future moments as prediction targets, which balances the model’s ability for long-term prediction and real-time inference efficiency.

In the sample filtering stage, abnormal incomplete samples with missing data, sudden trajectory jumps, or discontinuous timestamps within observation windows are automatically eliminated to ensure all training, validation, and test samples are complete and valid. Furthermore, to strengthen the model’s generalization ability and achieve spatial translation invariance, we abandon the direct input of absolute coordinates. Instead, global absolute position sequences are converted into inter-frame relative displacement sequences as network input. This processing method effectively weakens the negative influence of global airspace position bias on model training and makes the model concentrate more on local aircraft maneuver variations and temporal evolution rules.

The TrajAir dataset is partitioned at the trajectory level, where each complete raw ADS-B flight trajectory is treated as the fundamental splitting unit. Each flight is entirely allocated to the training, validation, or test partition, with a partition ratio of 60%/20%/20%. Sliding-window sampling is executed after trajectory-level division and is confined within each individual subset. Consequently, cropped samples derived from one original flight trajectory cannot appear across different partitions, eliminating temporal information leakage between training and test data.

### 4.2. Experimental Environment and Parameter Configuration

All experiments in this work are implemented on the PyTorch 2.2.1. deep learning framework, with model training and inference executed on an NVIDIA GeForce RTX 5070 graphics card (Nvidia Corporation, Santa Clara, CA, USA). Universal hyperparameters are predefined for all core network components: the dimension of hidden layers is fixed at 128, the number of attention heads is set to 8, two stacked layers are adopted for graph interaction modules, the latent space dimension is configured as 32, and the dropout probability is assigned 0.2.

For training configurations, each mini-batch contains 64 samples, and the training process terminates after 150 epochs at most. AdamW with weight decay serves as the optimization algorithm, and the weight decay factor is set to 10^−4^. We adopt a compound learning rate adjustment strategy combining linear warm-up and cosine annealing. The learning rate climbs slowly from 10^−5^ up to 10^−3^ in the warm-up period, and then decays to 10^−6^ for fine-tuning. This strategy delivers fast convergence at the beginning of training and precise parameter tuning in subsequent stages. In addition, gradient clipping with a threshold of 2.0 is adopted to suppress violent gradient oscillation during model updates.

When training multi-modal generative models, the KL divergence loss term often receives too little weight and fails to be fully optimized in the early training phase. To alleviate this problem, we implement an annealing strategy for the weight of the KL loss. The weight value rises linearly from 0 to 1 within the first 50 epochs. At the initial training stage, the model focuses mainly on trajectory reconstruction to quickly learn temporal variation rules and multi-aircraft spatial interaction features. As training enters the middle and later phases, regularization constraints on the latent distribution are gradually reinforced. This scheme stabilizes the training process and significantly boosts the quality of multi-modal trajectory generation.

### 4.3. Evaluation Metrics

To quantitatively evaluate the prediction precision and robustness of the proposed multi-modal trajectory prediction model, we adopt two standard metrics widely recognized in trajectory prediction research: average displacement error (ADE) and final displacement error (FDE). Both metrics are measured in kilometers (km), and they quantify spatial deviations between predicted flight paths and ground-truth trajectories via Euclidean distance calculations. Performance assessment is conducted from two separate dimensions: overall global fitting accuracy and terminal position forecasting precision. Together, they offer a comprehensive reflection of the model’s performance in long-horizon trajectory extrapolation tasks. ADE calculates the average spatial offset across all future prediction timestamps for every test sample. It objectively reflects the model’s overall fitting capacity for complete temporal trajectory evolution trends, serving as the core metric to measure global prediction performance. Its computational formula is written as follows:

(19) $$ADE=\frac{1}{N}\sum_{i=1}^{N}\frac{1}{T_{f}}{\sum_{t=1}^{T_{f}}\left\|y_{i}^{t}-y_{i_{GT}}^{t}\right\|}_{2}$$

where *N* denotes the total number of trajectory samples, $y_{i}^{t}$ represents the predicted position of the *i*-th trajectory at time step t, and $y_{i_{GT}}^{t}$ stands for the corresponding ground-truth position. A smaller ADE value indicates superior overall trajectory fitting precision of the model.

FDE quantifies the spatial discrepancy between the predicted terminal position and the ground-truth endpoint of a trajectory. It focuses on evaluating the model’s accuracy in forecasting aircraft’s long-range terminal flight positions, and carries important guiding significance for practical applications including airspace conflict detection and terminal airspace situation anticipation. The corresponding calculation formula is given below:

(20) $$FDE=\frac{1}{N}{\sum_{i=1}^{N}\left\|y_{i}^{T_{f}}-y_{i_{GT}}^{T_{f}}\right\|}_{2}$$

$y_{i}^{T_{f}}$ is the predicted position of the *i*-th trajectory at the final prediction time step $T_{f}$, and $y_{i_{GT}}^{T_{f}}$ is its matched ground-truth terminal coordinate. A lower FDE value signifies stronger long-range trajectory localization accuracy and situation prediction capability of the model.

### 4.4. Comparative Experiments

To comprehensively verify the effectiveness and superiority of the proposed model, eight representative trajectory prediction algorithms are chosen as baseline methods for comparative analysis. These baselines cover multiple technical categories, including traditional mechanism-driven approaches, classic machine learning algorithms, temporal deep learning networks, and spatial–temporal graph modeling schemes, enabling comprehensive performance evaluation from multiple dimensions. The selected comparative models include Const. Vel. [25], Nearest Neigh., SVM, LSTM [26], 1D-CNN [27], STG-CNN [28], TransformerTF [29], TrajAirNet [6] and ACTrajNet [1]. This benchmark encompasses heuristic physical baselines, conventional machine learning approaches, mainstream deep learning architectures for sequence and spatial–temporal modeling, as well as the CVAE-based multi-modal state-of-the-art TrajAirNet, which serves as the primary reference to verify the improvements brought by our newly introduced modules.

All baseline models are re-implemented and retrained under unified experimental configurations. They adopt the identical trajectory-level train-validation-test partition, data preprocessing procedure, length of historical observations, and prediction horizon. Note that intention-related and meteorological context representations are latent features derived within the internal IACEM of our model, rather than raw external inputs fed into the model’s input layer. All competing baseline methods take exactly the same raw kinematic trajectory observations (position, velocity, heading) as their only network input. Baseline models have no access to METAR meteorological records. Identical kinematic input guarantees fair quantitative comparison; performance improvements originate from our novel module architectures instead of privileged external input resources.

Quantitative results for all compared models evaluated on the TrajAir dataset are summarized in Table 1. Note that during offline quantitative evaluation, minADE and minFDE (best-of-20) are computed by selecting the sample closest to ground truth among 20 CVAE samples; this procedure uses future ground-truth and serves only for metric calculation as an upper-bound reference. For practical online deployment where ground-truth future trajectories are inaccessible, we perform clustering over all generated trajectory samples and adopt the centroid of the largest cluster as the final representative prediction.

Table 1 demonstrates that models adopting different modeling logics exhibit distinct performance gaps when tasked with forecasting multi-aircraft flight paths in non-towered terminal airspace. The conventional algorithms, including Const. Vel., Nearest Neigh. and SVM record much higher ADE and FDE scores, revealing poor prediction accuracy. The experimental results indicate that simple kinematic extrapolation or shallow machine learning regression cannot describe the highly non-linear variation in aircraft maneuvers under arrival, departure, and mutual avoidance conditions. Motion hypotheses assuming constant speed or acceleration cannot handle complex trajectory changes restricted by multi-aircraft coupling relations. Such traditional models can only support rough short-term prediction and fail to meet the requirement of precise long-horizon trajectory forecasting.

On the contrary, LSTM and 1D-CNN, two deep learning models that only process single-aircraft temporal data, achieve obvious error reduction. This proves that deep networks have prominent advantages in extracting temporal evolution features from trajectory sequences. However, these two models only analyze the time-series changes in a single aircraft’s historical track without explicitly modeling positional constraints and dynamic interactions among multiple aircraft. When dealing with dense traffic and close-range collision avoidance scenarios in terminal airspace, prediction errors caused by insufficient modeling information cannot be effectively eliminated, resulting in inherent limitations on model generalization.

The test results of STG-CNN and TransformerTF further prove that models overly biased toward spatial or temporal features cannot fully adapt to complex prediction scenes of terminal airspace. STG-CNN adopts graph structures to model multi-aircraft spatial correlations, but it relies on local convolution to construct temporal receptive fields, which limits its capacity to capture long-term trajectory trends and long-distance temporal dependencies. TransformerTF utilizes self-attention to strengthen long-sequence feature extraction, yet it does not explicitly characterize spatial interaction constraints such as physical separation and relative positions between aircraft. Both models have incomplete feature representation in either the spatial or temporal dimension.

In contrast, the baseline TrajAirNet, ACTrajNet, and our proposed model all perform joint modeling that fuses temporal evolution rules, multi-aircraft spatial interactions, and multi-source context information. These three methods achieve considerable error reduction across all four test subsets (7 Days-1 to 7 Days-4). ACTrajNet delivers moderate performance improvements over TrajAirNet by introducing altitude-oriented channel attention, yet it still underperforms our method. In particular, our model obtains the lowest ADE and FDE values on all four test datasets. This fully validates that our model can comprehensively capture long-term temporal evolution rules of aircraft trajectories, physical spatial constraints between multiple aircraft, and environmental prior information including meteorological factors and flight phases. Benefiting from this multi-dimensional fusion design, our approach achieves substantially improved adaptability and prediction performance for complex terminal airspace scenarios characterized by dense traffic, frequent maneuvers, and strong multi-aircraft coupling effects.

A horizontal spatial threshold of 600 m is adopted for constructing the dynamic adjacency matrix in ST-GAT. This value refers to practical horizontal separation guidelines for light-aircraft traffic-pattern operations at non-towered airports. Aircraft pairs whose horizontal Euclidean distance exceeds this threshold are removed from the graph, under the assumption that their mutual spatial interaction is negligible. This hyperparameter is optimized solely on the validation set and kept unseen from test-set data to prevent data leakage. It should be emphasized that the current graph-topology construction relies purely on horizontal separation, without taking relative velocity, heading, or vertical motion into account.

**Figure 4:**
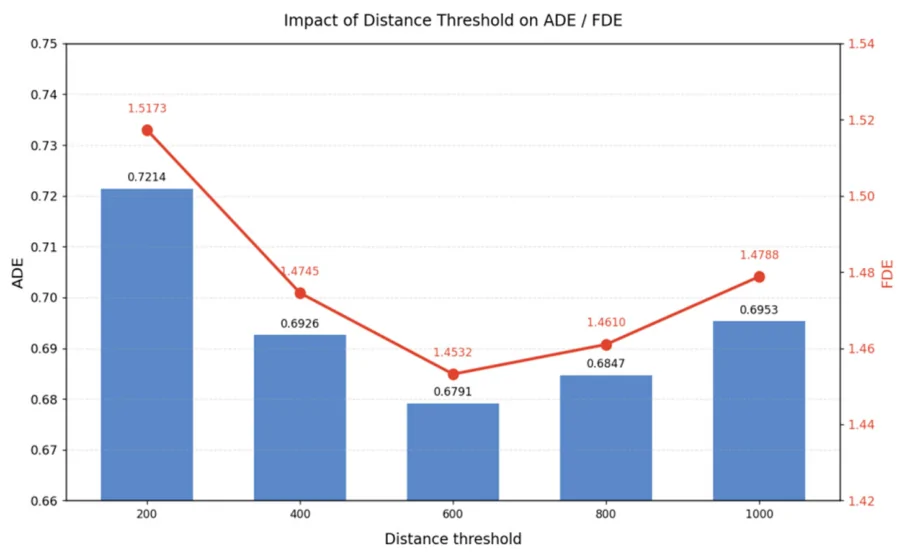
Sensitivity analysis of the spatial interaction threshold for ST-GAT on the Days-2 subset.

To further examine model robustness against this manually configured hyperparameter, we perform sensitivity analysis on the representative 7 Days-2 subset, with results visualized in Figure 4. A too-small threshold prunes functionally important interactive edges and hurts forecasting accuracy. By contrast, an overly large threshold creates noisy connections between spatially distant aircraft. Encouragingly, prediction performance remains stable across the moderate range of 400 m to 800 m. The chosen 600 m falls within this stable operating region, which confirms that the performance improvements of ST-GAT do not stem from a coincidental hyperparameter choice.

To mitigate the influence of training randomness, we train the proposed model and the dominant baseline TrajAirNet five times with different random seeds (10, 30, 50, 70, 90). For each metric, the mean ± standard deviation across repeated runs are reported as listed in Table 2.

We adopt the paired two-sided non-parametric Wilcoxon signed-rank test for statistical significance assessment. Each paired observation uses identical random seeds for TrajAirNet and the proposed model. Separate *p*-values are calculated for ADE and FDE, as reported in the footnote of Table 2. Statistically significant improvements *p* < 0.05 are observed for both ADE and FDE on 7 Days-1, 7 Days-2, and 7 Days-4. For the 7 Days-3 subset, FDE achieves a statistically significant improvement with *p* = 0.043, while ADE yields *p* = 0.061, which does not satisfy the α = 0.05 significance threshold. This marginal ADE outcome on 7 Days-3 is probably attributed to limited sample diversity in this particular test subset. Overall, most subsets demonstrate statistically valid performance gains beyond random training fluctuations.

### 4.5. Ablation Experiments

To quantify the independent contribution and cooperative effect of each innovative module on prediction accuracy, we carry out step-by-step ablation comparison experiments. The original TrajAirNet serves as the base network for comparison. This baseline model adopts TCN to extract temporal features and standard GAT to model graph interactions, lacking modules for flight intention discrimination and multi-source environmental context fusion.

We build a series of ablated model variants by adding three optimized components one by one to the baseline: ST-Transformer, physics-constrained ST-GAT, and IACEM intention-aware context fusion module. Table 3 presents the evaluation results of all ablation model groups on the test dataset.

A comparison between baseline Model A and Model B (only equipped with ST-Transformer) reveals that replacing the original TCN temporal encoder with global self-attention yields substantial FDE reductions across all four test datasets, which translates to remarkable improvements in terminal position forecasting accuracy. The underlying reason lies in the inherent limitations of TCN: it expands receptive fields merely through local convolutional kernels, making it incapable of capturing long-horizon trajectory evolution patterns spanning dozens of timestamps. In contrast, the ST-Transformer integrates global self-attention with temporal attention pooling. It directly establishes pairwise correlations between arbitrary timestamps within the full sequence, and adaptively screens critical historical maneuver segments that dominate long-term terminal positions. This design effectively mitigates cumulative prediction errors in long-sequence extrapolation. The experimental outcomes validate that self-attention-based temporal modeling delivers prominent gains for end-position prediction tasks.

Further stacking the physics-distance-aware ST-GAT upon Model B produces Model C, which achieves additional optimization in overall ADE metrics. Conventional vanilla GAT assigns interaction weights purely based on latent feature similarity, while ignoring physical separation constraints between aircraft. In dense multi-aircraft crossing scenarios, this easily triggers representation collapse and physically unrealistic trajectory overlapping artifacts. The ST-GAT incorporates explicit Euclidean physical distance encoding; grounded in airspace safety separation priors, it automatically allocates higher interaction weights to nearby neighboring aircraft and suppresses modeling noise introduced by distant, irrelevant agents. The injection of physical priors standardizes the logic of multi-aircraft interaction representations, drastically enhancing the spatial rationality of predicted flight paths in crowded terminal airspace, and noticeably cutting the global average trajectory offset reflected by ADE.

To reiterate, adding ST-GAT to Model B (Model C) further lowers ADE values. The original GAT relies solely on latent feature similarity and tends to suffer from representation collapse. By embedding explicit Euclidean distance encoding, ST-GAT compels the model to assign larger attention weights to physically adjacent aircraft. The introduction of physical priors effectively alleviates trajectory crossing artifacts in dense traffic scenes and significantly boosts the spatial plausibility of generated trajectories.

After integrating the IACEM, our complete model achieves the optimal ADE and FDE simultaneously on all four test subsets (7 Days-1 to 7 Days-4), with both metrics reaching their minimum values. This fully demonstrates that high-level flight operational intentions and multi-source contextual signals (meteorological conditions, airspace traffic status, etc.) serve as critical constraints governing trajectory evolution. Spatio-temporal motion features alone cannot fully reconstruct the genuine maneuver decision-making logic of aircraft. The IACEM module first completes flight-phase intention recognition using historical temporal embeddings, then dynamically fuses heterogeneous environmental information via adaptive attention. It supplies the model with complete high-level semantic and environmental conditional inputs, compensating for the lack of contextual priors in the previous two ablated variants.

Comprehensive gradient comparison results across the three groups of ablation models lead to the following conclusion: ST-Transformer, ST-GAT, and IACEM each deliver distinct performance improvements to the baseline model from three orthogonal dimensions: mining long-range temporal dependencies, characterizing physical multi-aircraft interactions, and fusing multi-source context with flight intentions. The three modules provide mutually complementary information. When deployed collaboratively, they enable complete joint modeling of temporal evolution, spatial physical constraints, and environmental semantic information, maximizing the overall prediction accuracy and robustness for multi-aircraft trajectory prediction in non-towered terminal airspace.

In addition to the incremental additive ablation (Model A → B → C → Ours), we further conduct leave-one-out experiments based on the full model, where each key module is removed individually while keeping the other two components intact. As shown in Table 4, removing any single module consistently degrades prediction performance. The performance drop is most prominent when ST-GAT is excluded, indicating its critical role in capturing multi-aircraft spatial interactions. ST-Transformer and IACEM also yield non-negligible individual gains within the complete framework. These observations demonstrate that the three modules provide complementary functionality for multi-aircraft trajectory prediction.

**Figure 5:**
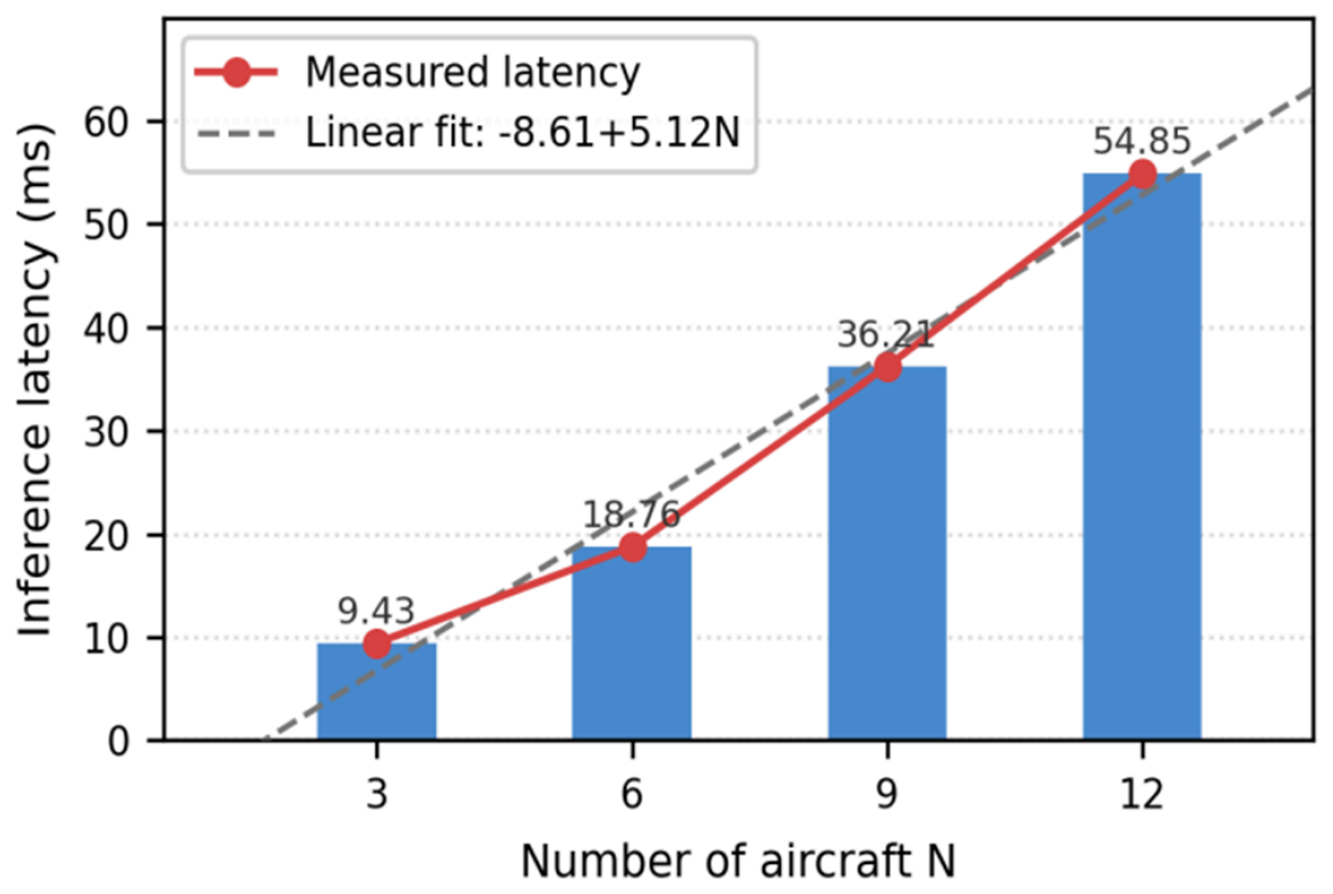
The inference latency results for the number of aircraft N.

Moreover, computational overhead is evaluated on a GeForce RTX 5070 GPU. The model has 12.46 M trainable parameters. Figure 5 shows the measured inference latency for *N* = {3,6,9,12}. Benefiting from the dynamically built adjacency matrix in ST-GAT, attention operations are restricted to spatially proximate aircraft. The system avoids the *O*(*N*^2^) cost of full pairwise attention. A mild upward tendency can be observed as *N* grows, originating from GPU memory access overhead, yet no quadratic complexity explosion is observed. Even for *N* = 12, the inference latency remains 54.85 ms, supporting real-time conflict early-warning.

### 4.6. Visualization Analysis

Aircraft trajectory evolution within terminal airspace is governed by coupled constraints stemming from multiple factors: each aircraft’s historical maneuver states, dynamic interactions with surrounding planes, meteorological variations, and distinct flight operation phases. Such complex coupled effects inherently bring prominent multi-modal uncertainty to predicted future flight paths.

**Figure 6:**
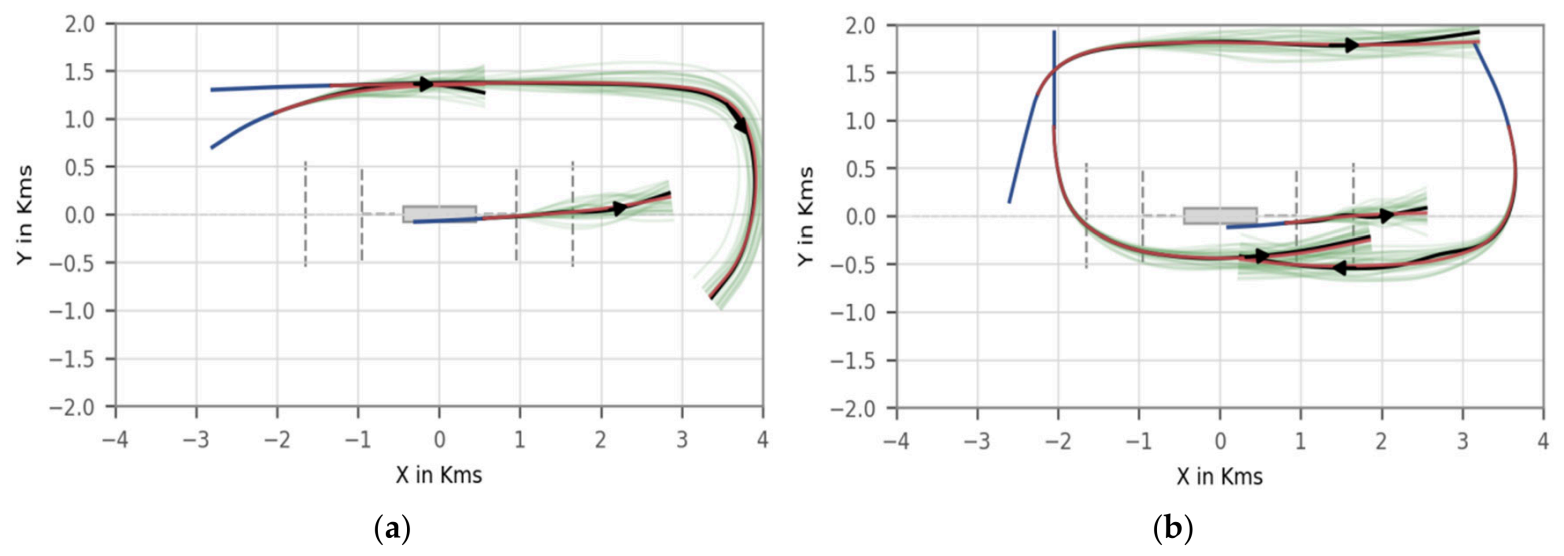
Trajectory prediction results under multi-aircraft interaction scenarios. (**a**) Three-aircraft interaction on the downwind leg of the airport traffic pattern, and (**b**) four-aircraft interaction within the closed-loop airport traffic pattern.

Multiple aircraft frequently perform coordinated maneuvers, including speed adjustments for collision avoidance and cooperative route modifications. Compared with single-aircraft flight scenarios, such interactive behaviors broaden the fluctuation range of trajectory distributions. Accordingly, higher requirements are imposed on the model to capture inter-aircraft spatial constraints as well as their coordinated temporal evolution patterns. Figure 6 visualizes the predicted trajectories for two representative multi-aircraft interaction scenarios within the airport traffic pattern. The blue line represents the historical observation trajectory, the red line represents the ground-truth trajectory, and the green semi-transparent area represents the multi-modal sampling distribution output by the proposed model. The black dashed line represents the post-optimal sample with the smallest deviation from the true value space. Note that this sample was obtained through offline screening of truth values after sampling and is only used for visual display. It is not an autonomous output result during the model inference stage.

Figure 6a illustrates a three-aircraft cooperative interaction on the downwind leg of the airport traffic pattern. Two aircraft maintain close-parallel flight on two parallel tracks of the downwind segment, while the third aircraft performs a high-curvature turn toward the base leg at the right end of the airspace. In terms of trajectory morphology, the multi-modal sampled trajectories fit well with the red ground-truth trajectories. The model delivers precise predictions of the turning curvature and terminal turning position of the aircraft on the right. The predicted paths of aircraft on the upper and lower parallel tracks exhibit only minor deviations, with compact multi-modal distribution ranges. No unrealistic artifacts are observed, such as abnormally reduced separation between adjacent aircraft or intersecting trajectories that violate airspace safety separation standards. Combined with the quantitative metrics listed in Table 3, the proposed model reduces ADE by 10.38% and FDE by 8.31% compared with the baseline model under the three-aircraft scenario. This verifies that the physical distance constraints embedded in the ST-GAT module can reliably regularize the relative arrangement of closely spaced aircraft and prevent physically implausible spatial position predictions.

Figure 6b depicts a four-aircraft interaction case within a full closed-loop airport traffic pattern. Aircraft are distributed over downwind leg, base leg, and final-approach leg segments of the circuit, bringing multiple local route intersections and successive turning sections. More mutually restrictive collision-avoidance maneuvers occur between adjacent aircraft, leading to higher spatial-interaction complexity compared with the downwind-leg-only scenario in Figure 6a. From the visualization perspective, the model accurately captures the overall circular route layout. The curvature variations across all continuous turning segments and maneuver transition trends at intersection points align well with ground-truth trajectories. Superimposed restrictive effects from multiple surrounding aircraft increase uncertainty in flight decision-making, which widens the dispersion of sampled trajectories in local areas relative to the three-aircraft scenario. Such broader distribution is a reasonable characteristic of multi-modal prediction, without large-scale trajectory deviations. Referring to the evaluation metrics in Table 5, both models show slight error increases in response to higher interaction complexity. Nevertheless, our proposed model still achieves a 9.64% reduction in ADE and a 9.57% reduction in FDE. As the number of interacting aircraft increases, the baseline model suffers a more dramatic error surge. Benefiting from joint regularization provided by the physics-aware graph attention and intention-context fusion modules, our model effectively suppresses error growth when handling complex coupled airspace interactions, demonstrating further enhanced robustness advantages.

**Figure 7:**
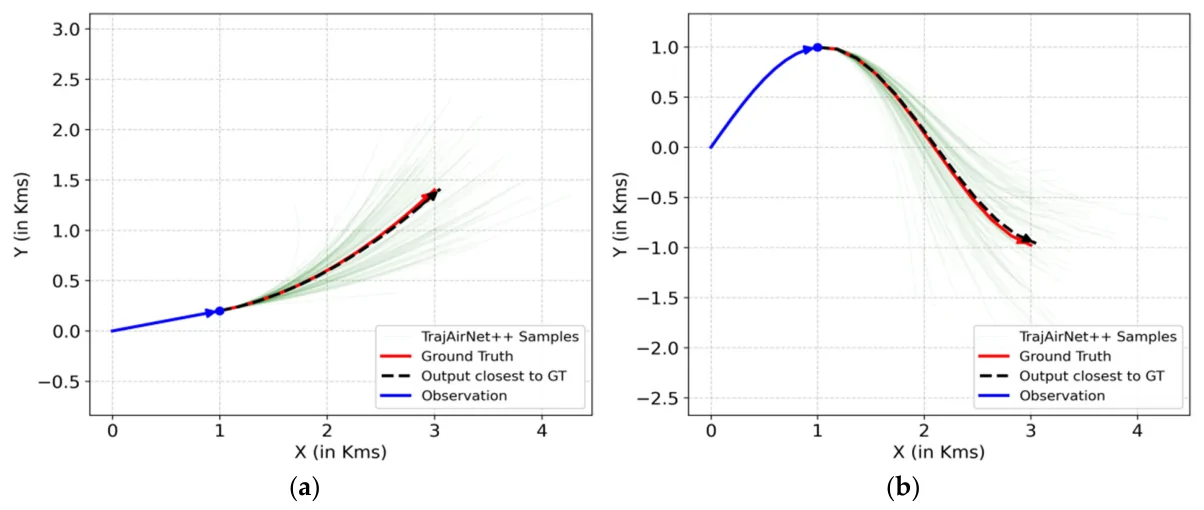

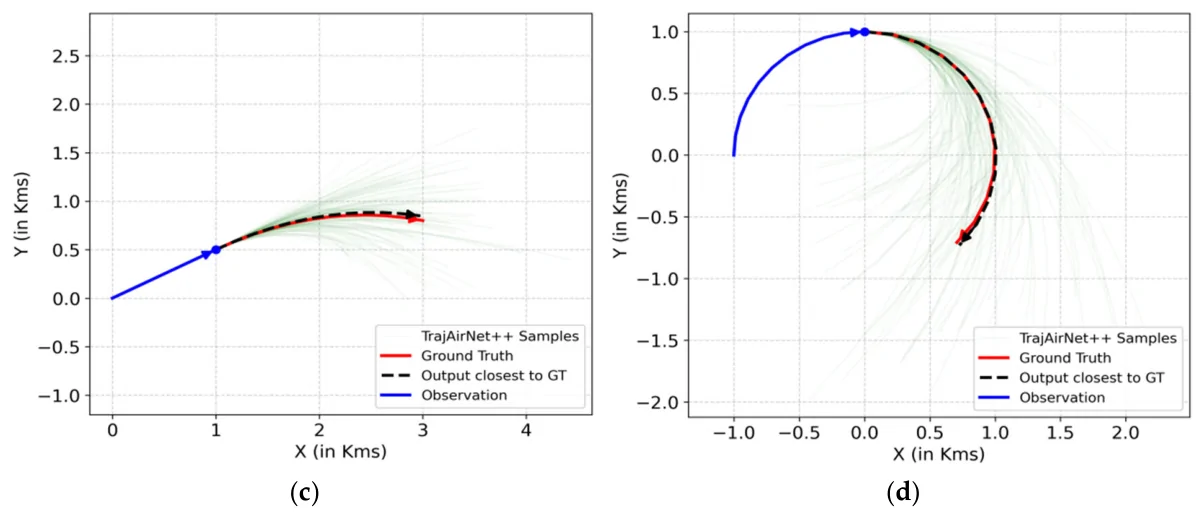
Visualized trajectory outputs under various typical maneuver conditions. (**a**) Gentle turns. (**b**) S-shaped maneuvers. (**c**) Straight flight with right turns. (**d**) sharp U-turns.

To intuitively verify the prediction and generation performance of the proposed model under various representative maneuver conditions, this section assesses the morphological smoothness and physical airspace rationality of predicted flight paths from two perspectives: spatial dispersion of trajectory distribution and temporal evolution trends. Figure 7 displays two-dimensional visualized trajectory results for four typical flight maneuvers, namely gentle turns, S-shaped maneuvers, straight flight with right turns, and sharp U-turns. In the figure, solid blue lines stand for historically observed trajectories, solid red lines denote ground-truth future flight paths, green semi-transparent bands represent multi-modal sampling distributions generated by our proposed model, and black dashed lines mark the prediction sample with the minimal offset from the ground truth. Note that during offline quantitative evaluation, minADE and minFDE (best-of-20) are computed by selecting the sample closest to the ground truth among 20 CVAE samples; this procedure uses future ground-truth and serves only for metric calculation. For practical online deployment where ground-truth future trajectories are inaccessible, we perform clustering over all generated trajectory samples and adopt the centroid of the largest cluster as the final representative prediction, without relying on any future reference information. It can be observed that the multi-modal trajectories generated by the model under all four conditions feature smooth overall contours. The extending tendency of sampled regions closely matches the maneuver variation patterns of real flight paths, without unrealistic artifacts such as abrupt bends or directional misalignment that violate the physical constraints of aircraft motion.

For low-maneuver-intensity cases, including the gentle turn in Figure 7a and straight flight with a right turn in Figure 7c, predicted trajectories extend steadily along the real flight direction with compact modal distribution and low dispersion. This demonstrates that the model can accurately capture the temporal evolution patterns underlying steady maneuvers with minor constant-speed adjustments. In contrast, the S-shaped maneuver in Figure 7b and sharp U-turn in Figure 7d constitute challenging scenarios with drastic heading shifts, where aircraft experience large dynamic directional deflections and markedly strengthened non-linear maneuver characteristics. Despite the elevated difficulty of these working conditions, all sampled trajectories are still capable of reproducing core turning tendencies, and the spatial deviation between the optimal prediction sample and ground-truth trajectory remains maintained at a low level.

**Figure 8:**
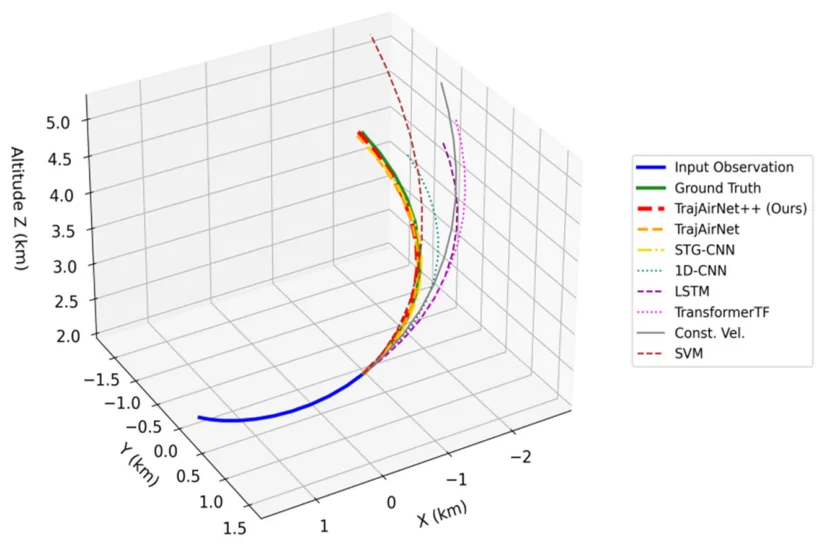
Comparison of predicted trajectories from different models in the 3D climbing and circling scenario.

Aircraft arrival and departure operations within terminal airspace are frequently accompanied by drastic altitude variations along the *Z*-axis. Accordingly, comparative prediction experiments of all models are carried out for a 3D climbing-and-loitering scenario, with corresponding results illustrated in Figure 8. Obvious performance gaps can be observed among different baseline models in this climbing loitering case. SVM and constant velocity models fail to characterize sophisticated non-linear flight maneuvers, leading their predicted trajectories to diverge from ground-truth paths at an early stage. While LSTM and TransformerTF can retain basic temporal continuity, they still exhibit noticeable biases when jointly modeling altitude shifts and turning procedures. The baseline TrajAirNet roughly follows the overall flight tendency yet suffers outward expansion errors at the tail segment of trajectories.

In comparison, the three-dimensional flight paths generated by our proposed model overlap closely with ground-truth references throughout the entire prediction horizon. The model achieves high-precision fitting for both horizontal turning routes and vertical altitude ascent/descent profiles. This outstanding performance stems from the explicit integration of airspace physical distance priors within the ST-GAT module. Unlike conventional GAT architectures that assign interaction weights merely based on latent feature similarity, ST-GAT incorporates Euclidean distances between aircraft into the attention scoring pipeline. It adaptively distributes interaction weights for neighboring aircraft in compliance with terminal airspace safety separation regulations. This design effectively restrains three-dimensional spatial drift under coupled multi-aircraft interactions and greatly improves prediction accuracy for complex 3D maneuver trajectories such as climbing loitering flights.

Experimental results fully verify that the three core innovative modules—the ST-Transformer for long-range temporal modeling, the physics-distance-aware ST-GAT for spatial interaction constraints, and the intention-aware IACEM for multi-source context fusion—can each cut prediction errors independently and deliver prominent positive synergistic gains when combined. Joint modeling with all three modules enables simultaneous extraction of aircraft temporal evolution patterns, physical spatial constraints among multiple aircraft, and flight semantic environmental information, thoroughly remedying the modeling deficiencies inherent to existing baseline models.

Taken together, all experimental evidence comprehensively demonstrates that the multi-modal trajectory prediction algorithm proposed in this paper achieves reliable effectiveness and remarkable performance superiority when applied to multi-aircraft interaction prediction within dense, non-towered terminal airspace.

Besides ADE and FDE for trajectory-fitting evaluation, two safety-oriented metrics are introduced to assess spatial-separation properties of multi-aircraft predicted trajectories, namely average minimum inter-aircraft horizontal distance and separation-violation rate. The average minimum inter-aircraft horizontal distance denotes the minimal horizontal distance between any pair of aircraft throughout each predicted sequence, where higher values represent better-maintained spatial separation. The separation-violation rate refers to the proportion of test samples where the minimum pairwise horizontal distance drops below the 600 m safety threshold defined for non-towered airport scenarios, and a lower rate indicates fewer unsafe prediction outputs. Note that these metrics characterize the spatial behavior of predicted trajectories and do not equal the real-world conflict-detection performance of an operational system.

Table 6 summarizes the safety-related results on the 7 Days-2 subset. Completely ignoring multi-aircraft interactive constraints, the Const. Vel. baseline achieves an average minimum distance of 427.31 m, with a separation-violation miss rate of 27.38% and a false-alarm rate of 22.84%. LSTM and STG-CNN obtain moderate performance, with average minimum distances of 486.75 m and 532.48 m, miss rates of 22.09% and 18.57%, and false-alarm rates of 18.30% and 15.76%, respectively. As a strong multi-modal baseline, TrajAirNet yields average minimum distances of 574.26 m, a miss rate of 14.16%, and a false-alarm rate of 12.37%. Benefiting from physics-aware spatial regularization in ST-GAT, our model delivers the best overall safety performance: an average minimum inter-aircraft distance of 628.53 m, alongside a miss rate of 8.34% and a false-alarm rate of 7.62%. Notably, the larger average minimum distance is accompanied by reduced miss and false-alarm rates, proving that our model does not merely overestimate aircraft separation. These results confirm that ST-GAT suppresses unrealistic close-range spatial layouts and reproduces realistic inter-aircraft proximity patterns.

## 5. Conclusions

Further comparison against closely related approaches highlights our technical differences. Pure Transformer-based trajectory models typically deploy global self-attention without physical-constraint embedding. Most physics-aware graph prediction methods rely on static graph structures and apply physical rules only in loss functions. Existing intention-aware or goal-conditioned predictors commonly need pre-estimated flight destinations as model input. By contrast, our ST-Transformer, ST-GAT, and IACEM respectively address these limitations: physical constraints are embedded inside attention computation; graph topology is dynamically updated by aircraft spatial distribution; and implicit intention is inferred without explicit destination priors. The core contributions and key findings are summarized as follows:

(1) To overcome the limited receptive field of TCN and its poor performance in capturing long-range temporal dependencies, this work combines global self-attention with temporal attention pooling to extract trajectory evolution patterns across all time steps. This design mitigates error accumulation during long-term prediction and yields notable reductions in FDE.
(2) Conventional GAT layers assign interaction weights solely based on latent feature similarity, which often leads to physically unrealistic trajectory crossing and spatial drift among multiple aircraft. This paper embeds Euclidean distance encoding between aircraft into the attention calculation pipeline. Higher interaction weights are automatically assigned to aircraft with close spatial proximity, which regularizes the relative spatial layout of multiple aircraft. This mechanism steadily cuts the global ADE and alleviates trajectory divergence in highly coupled 3D scenarios such as climbing circling flights.
(3) An auxiliary branch for flight intention recognition is incorporated, which leverages cross-entropy loss to classify different aircraft maneuver types. Adaptive attention is adopted to dynamically fuse METAR meteorological parameters, compensating for missing environmental and semantic constraints that cannot be captured by pure spatio-temporal modeling.

Overall, the proposed method partially addresses several key deficiencies of prior approaches in temporal, spatial, and contextual modeling for non-towered terminal-airspace scenarios, achieving encouraging prediction accuracy and robustness within the TrajAir dataset test cases. It shows promising adaptability to scenarios with dense traffic, complex flight maneuvers, and tight multi-aircraft coupling. The above observations are limited to the evaluated dataset and do not represent universal performance for all airspace environments.

Limitations of this work should also be noted. All experiments are carried out on trajectory data from a single airport. Variations in airport geometry, runway layout, and local traffic-pattern procedures will induce distribution shift. Therefore, the cross-airport generalization of the proposed model has not been fully validated. Sufficient publicly available multi-site non-towered-airport trajectory datasets are lacking. Future work will extend the model toward multi-airport datasets, enrich physical modeling by integrating relative velocity, heading, and vertical convergence metrics, and optimize the model for lightweight embedded deployment to support practical conflict early-warning applications.

## Figures and Tables

**Table 1:** The quantitative metric results of all comparison models tested on the TrajAir dataset.

Model	7 Days-1		7 Days-2		7 Days-3		7 Days-4	
	ADE	FDE	ADE	FDE	ADE	FDE	ADE	FDE
Const. Vel.	1.7976	4.0853	1.9029	4.3187	1.9241	4.3035	1.8195	4.1638
Nearest Neigh.	2.1312	2.7088	1.9247	1.9916	2.4135	2.6947	1.5923	2.5759
SVM	2.6491	4.8846	2.7233	5.1175	2.8017	5.1548	2.5792	4.8534
LSTM	1.6184	3.7519	1.7543	3.9826	1.8139	4.1191	1.5965	3.8247
1D-CNN	1.4837	3.4190	1.5462	3.6614	1.6188	3.7535	1.4541	3.3872
STG-CNN	1.1658	2.3124	1.3339	2.6317	1.3146	2.6185	1.1530	2.2497
TransformerTF	1.5173	3.7045	1.6321	3.9528	1.8849	4.2236	1.7170	4.0545
TrajAirNet	0.7266	1.4094	0.7938	1.6155	0.8472	1.7113	0.6945	1.3881
ACTrajNet	0.7082	1.3741	0.7734	1.5782	0.8246	1.6695	0.6728	1.3494
Ours	0.6420	1.2776	0.6791	1.4532	0.7245	1.4589	0.6516	1.2843

**Table 2:** Statistical performance comparison over repeated training runs on four 7-Day subsets.

	Evaluation Metrics	TrajAirNet	Ours
7 Days-1	ADE	0.7266 ± 0.0221	0.6420 ± 0.0183
	FDE	1.4094 ± 0.0415	1.2776 ± 0.0342
7 Days-2	ADE	0.7938 ± 0.0247	0.6791 ± 0.0205
	FDE	1.6155 ± 0.0483	1.4532 ± 0.0394
7 Days-3	ADE	0.8472 ± 0.0284	0.7245 ± 0.0236
	FDE	1.7113 ± 0.0526	1.4589 ± 0.0448
7 Days-4	ADE	0.6945 ± 0.0196	0.6516 ± 0.0168
	FDE	1.3881 ± 0.0372	1.2843 ± 0.0315

**Table 3:** The evaluation results of all ablation model groups on the test dataset.

Model Variant	ST-Transformer	ST-GAT	IACEM	7 Days-1		7 Days-2		7 Days-3		7 Days-4	
				ADE	FDE	ADE	FDE	ADE	FDE	ADE	FDE
Model A (TrajAirNet)	—	—	—	0.7266	1.4094	0.7938	1.6155	0.8472	1.7113	0.6945	1.3881
Model B	✓	—	—	0.7052	1.3917	0.7625	1.5743	0.8164	1.6692	0.6731	1.3568
Model C	✓	✓	—	0.6978	1.3146	0.7319	1.5287	0.7335	1.6247	0.6604	1.3015
Ours (TrajAirNet++)	✓	✓	✓	0.6420	1.2776	0.6791	1.4532	0.7245	1.4589	0.6516	1.2843

**Table 4:** Ablation results of the proposed modules.

Model Variant	7 Days-1		7 Days-2		7 Days-3		7 Days-4	
	ADE	FDE	ADE	FDE	ADE	FDE	ADE	FDE
w/o ST-Transformer	0.6714	1.3082	0.7086	1.4841	0.7533	1.4926	0.6782	1.3127
w/o ST-GAT	0.6883	1.3357	0.7247	1.5164	0.7726	1.5304	0.6894	1.3386
w/o IACEM	0.6978	1.3146	0.7319	1.5287	0.7335	1.6247	0.6604	1.3015
Ours	0.6420	1.2776	0.6791	1.4532	0.7245	1.4589	0.6516	1.2843

**Table 5:** Evaluation metric results under different multi-aircraft interaction scenarios.

Interaction Scenario	Model	ADE	FDE
Three-aircraft interaction	TrajAirNet	0.7012	1.3647
Three-aircraft interaction	Ours	0.6284	1.2513
Four-aircraft interaction	TrajAirNet	0.7865	1.5329
Four-aircraft interaction	Ours	0.6907	1.3862

**Table 6:** The safety-related results on the 7 Days-2 subset.

Model	Avg. Min Distance (m)	Separation-Violation Miss Rate (%)	False-Alarm Rate (%)
Const. Vel.	427.31	27.38	22.84
LSTM	486.75	22.09	18.30
STG-CNN	532.48	18.57	15.76
TrajAirNet	574.26	14.16	12.37
Ours	628.53	8.34	7.62

## Data Availability

The original contributions presented in this study are included in the article. Further inquiries can be directed to the corresponding author.

## References

[1] Zhu H., Tong Q., Hu J., Liu X., Hou S. (2025). Altitude aware trajectory prediction methods for non towered terminal airspace. Sci. Rep..

[2] Aircraft Owners and Pilots Association (2003). Operations at Non-Towered Airport.

[3] Chen Y., Xu Y., Yang L., Hu M. (2025). In-flight fast conflict-free trajectory re-planning considering UAV position uncertainty and energy consumption. Transp. Res. Part C Emerg. Technol..

[4] Ge C., Zhang J., Wang L., Wang X., Yao J., Yang T., Du J. (2026). PC-Transformer: Probabilistic flight trajectory prediction with multi-stage predictive coding. Transp. Res. Part C Emerg. Technol..

[5] Yang S., Liu L., Chen B., Cheng S., Shi Z., Zou Z. (2025). GooDFlight: Goal-Oriented Diffusion Model for Flight Trajectory Prediction. IEEE Trans. Aerosp. Electron. Syst..

[6] Patrikar J., Moon B., Oh J., Scherer S. (2022). Predicting like A Pilot: Dataset and Method to Predict Socially-Aware Aircraft Trajectories in Non-Towered Terminal Airspace. 2022 IEEE International Conference on Robotics and Automation (ICRA).

[7] Lin C., Kau L., Chan C. (2022). Bimodal Extended Kalman Filter-Based Pedestrian Trajectory Prediction. Sensors.

[8] Ahmad E., He Y., Luo Z., Lv J. (2024). A Hybrid Long Short-Term Memory and Kalman Filter Model for Train Trajectory Prediction. IEEE Trans. Intell. Transp. Syst..

[9] Qiao S., Shen D., Wang X., Han N., Zhu W. (2015). A Self-Adaptive Parameter Selection Trajectory Prediction Approach via Hidden Markov Models. IEEE Trans. Intell. Transp. Syst..

[10] Chen N., Sun Y., Wang Z., Peng C. (2022). Correction and Fitting Civil Aviation Flight Data EGT Based on RPM: Polynomial Least Squares Analysis. Appl. Sci..

[11] Gao J., Dang Y., Zhu Y., Xue W. (2025). Moving-Target Tracking in Airport Airside Operations Using AIMM-STUKF. Sensors.

[12] Wang T., Skulstad R., Li G., Zhang H. (2026). An Interactive Multiple-Model Approach for Accurate and Interpretable Trajectory Prediction in Autonomous Docking. IEEE Trans. Autom. Sci. Eng..

[13] Zhang Z., Zhang J., Lin Y., Zhang K., Zheng X., Xiang D. (2025). Spatial-Temporal Physics-Constrained Multilayer Perceptron for Aircraft Trajectory Prediction. Appl. Sci..

[14] Rohani A.S., Puranik T.G., Kalyanam K.M. Machine Learning Approach for Aircraft Performance Model Parameter Estimation for Trajectory Prediction Applications. Proceedings of the 2023 IEEE/AIAA 42nd Digital Avionics Systems Conference (DASC).

[15] Song K., Li X., Wu Q. (2026). A CNN-LSTM-Based Multivariate Time Series Model for Aircraft Trajectory Prediction. Int. J. Aerosp. Eng..

[16] Xie G., Shangguan A., Fei R., Ji W., Ma W., Hei X. (2020). Motion trajectory prediction based on a CNN-LSTM sequential model. Sci. China Inf. Sci..

[17] Shi Z., Xu M., Pan Q. (2021). 4-D Flight Trajectory Prediction with Constrained LSTM Network. IEEE Trans. Intell. Transp. Syst..

[18] Rossi L., Paolanti M., Pierdicca R. (2021). Human trajectory prediction and generation using LSTM models and GANs. Pattern Recognit..

[19] Gu X., Wang J., Cheng D., Li C., Huang Q. (2024). Efficient multi-target vehicle trajectory prediction based on multi-scale graph convolution. Pattern Anal. Appl..

[20] Xu Z., Liu Y., Chu X., Tan X., Zeng W. (2023). Aircraft Trajectory Prediction for Terminal Airspace Employing Social Spatiotemporal Graph Convolutional Network. J. Aerosp. Inf. Syst..

[21] Wang J., Liu K., Li H., Gao Q., Wang X. (2025). Vehicle Trajectory Prediction Using Hierarchical LSTM and Graph Attention Network. IEEE Internet Things J..

[22] Zhang H., Liu Z. (2024). Four-Dimensional Aircraft Trajectory Prediction Based on Generative Deep Learning. J. Aerosp. Inf. Syst..

[23] Chen W., Sang H., Wang J., Zhao Z. (2024). DSTCNN: Deformable spatial-temporal convolutional neural network for pedestrian trajectory prediction. Inf. Sci..

[24] Gao Z., Yi W. (2026). Trajectory prediction method for incoming guided projectiles based on the fusion of Temporal Convolutional Network and dual attention mechanisms. Measurement.

[25] Schöller C., Aravantinos V., Lay F., Knoll A. (2020). What the Constant Velocity Model Can Teach Us About Pedestrian Motion Prediction. IEEE Robot. Autom. Lett..

[26] Zhang C., Yang P., Liang Y., Zhu B., Zhu W. (2024). Real-time track prediction of air combat UAV based on LSTM. Tactical Missile Technol..

[27] Alturif G., El-Bary A., Osman R. (2025). A deep learning approach to gender equality: Forecasting educational indicators with 1D-CNN aligned with SDG 5. PLoS ONE.

[28] Mohamed A., Qian K., Elhoseiny M., Claudel C. (2020). Social-stgcnn: A social spatio-temporal graph convolutional neural network for human trajectory prediction. Proceedings of the 2020 IEEE/CVF Conference on Computer Vision and Pattern Recognition (CVPR).

[29] Giuliari F., Hasan I., Cristani M., Galasso F. (2021). Transformer Networks for Trajectory Forecasting. 25th International Conference on Pattern Recognition (ICPR).

